# Bioethics and Biopolitics: Presents and Futures of Reproduction

**DOI:** 10.1007/s11673-017-9787-8

**Published:** 2017-06-12

**Authors:** Silvia Camporesi

**Affiliations:** Department of Global Health & Social Medicine, School of Global Affairs, King’s College London, D6, 2nd floor, East Wing, London, WC2R 2LS UK

**Keywords:** Reproduction, Biopower, Ectogenesis, Preimplantation genetic diagnosis, Reproductive rights, Pregnancy

## Abstract

This *Bioethics and Biopolitics: Presents and Futures of Reproduction* symposium draws together a series of articles that were each submitted independently by their authors to the *JBI* and which explore the biopower axis in the externalization of reproduction in four contexts: artificial gestation (ectogenesis), PGD for sex selection**,** women’s (reproductive) rights**,** and testicular cryopreservation (TCCP). While one contribution explores a “future” of reproduction, the other three explore a “present,” or better, explore different “presents.” What may counts as “present,” and what may count as “future,” has dramatically different connotations depending on the geographical declination of the tense.

## Introduction

Human reproduction is increasingly being externalized. We have a number of technologies that make this possible: in vitro fertilization (IVF), preimplantation genetic diagnosis (PGD), gamete donation, surrogacy, and, more recently, mitochondrial transfer technologies and uterus transplant.

This externalization goes hand in hand with an increasing commercialization of human reproduction which, in turn, goes hand in hand with an unprecedented responsibilization of reproduction whereby individuals become “managers” of their pregnancies. Nowadays, this is one of main axes of biopower and biopolitical management, where “individuals themselves are responsible for the enactment of biopolitics in reproduction” (Mills 2015, 112).

This *Bioethics and Biopolitics: Presents and Futures of Reproduction* symposium draws together a series of articles that were each submitted independently by their authors to the *JBI* and which explore the biopower axis in the externalization of reproduction in four contexts: artificial gestation (ectogenesis), PGD for sex selection**,** women’s (reproductive) rights**,** and testicular cryopreservation (TCCP). While one contribution explores a “future” of reproduction, the other three explore a “present,” or better, explore different “presents.” What may counts as “present,” and what may count as “future,” has dramatically different connotations depending on the geographical declination of the tense.

## Presentation of Contributions

The paper “The Perfect Womb: Promoting Equality of (Fetal) Opportunity” by Kendal (2017) discusses “partial ectogenesis”—defined as the ability to grow the fetus outside of a woman’s body for part of the fetal development. Kendal notes how partial ectogenesis is already routinely practiced in neonatology. At the one end of gestation we are now able to culture embryos in vitro for longer (up to 11–12 days) (Deglincerti et al. 2016; Shahbazi et al. 2016), while at the other end of gestation, progresses in perinatalogy have been pushing the limit for viability of prematurely born babies down from twenty-four weeks to twenty-three to twenty-two (Mercer 2017). The paper by Kendal points to an “elephant in the room,” i.e. that in the ongoing discussions of moving the limit on embryo research beyond fourteen days and of germline genome editing (Cavaliere 2017; Hyun 2016) nobody seems to be talking about ectogenesis. But ectogenesis is truly the elephant in the room: if we did not have this limit, research could continue beyond fourteen days to explore ways in which an embryo can be sustained in vitro.

As a matter of fact, this is not research that is impossible to do (as studies in other species show), but it is simply research that is not done. Why is that the case? Although the paper by Kendal cannot explore this further, it seems to indicate that it might be a “taboo” subject in research, where there are so many other ethical “battles” to fight (think of embryo research or of the renewed conflict around women’s reproductive rights). Perhaps the battle to liberate women from their biology is not one that raises the appetite of many, including funders, in a system of scarce resources where there are more “pressing” issues to attend. The reading that ectogenesis could finally “empower” women with a final decoupling of reproduction from biology is also, one should note, a partial one. Another reading could be that ectogenesis could further “discipline women” along the lines of the ways in which IVF, oocyte preservation, and other technological means of delaying pregnancy without compromising the optimality of the process are now disciplining and “responsibilizing” women.

The paper by Browne (2017), “How Sex Selection Undermines Reproductive Autonomy,” examines how sex selection through PGD may not enhance but rather undermine the scope of reproductive autonomy. This paper is particularly interesting as it conceptualizes autonomy in two ways: a) option and decision heavy (focused on a range of choices) and b) ultimate goal heavy (maximizing one’s freedom to achieve a goal). Kendal argues that sex selection, while enhancing the first kind of autonomy, undermines the second because it derives its existence from gender essentialism, i.e. the belief “that one cannot enjoy the same activities or have the same kind of relationship with a boy as with a girl (or vice versa)” (Kendal 2017, 3). Kendal builds directly on the work by feminist philosopher Catherine Mills and her inquiry into biopower in the context of reproduction (Mills 2011, 2015).

Kendall argues that we should move away from an understanding of autonomy in reproduction from an “option and decision heavy” model, towards an “ultimate goal heavy” understanding of autonomy. In Mills’ words (2011), we should move away from a negative understanding of reproductive freedom as “freedom from” only (building on Robertson 1983, 2004) to a positive understanding as “freedom to,” which includes the possibility to shape oneself while shaping others.

The paper by Princewill et al. (2017) “Autonomy and Reproductive Rights of Married Ikwerre Women in Rivers State, Nigeria” is a qualitative paper investigating how women in the Ikwerre community in Nigeria conceptualize reproductive rights and autonomy in marriage. Princewill et al. set out to examine two things: a) how married Ikwerre women understand reproductive rights and autonomy and b) what affects the exercise of their reproductive rights and of their autonomy within their marriage (mainly economic and educational status). Not surprisingly, the results of the study are that the majority of these women have no knowledge or very limited knowledge of reproductive rights, while they have a “fairly” developed knowledge of what autonomy meant, even though the exercise of autonomy was often not perceived as being “appropriate” for a woman. Princewill and colleagues argue that their study supports “creating awareness” among women of their reproductive rights, increasing “education” to ensure “empowerment” and promote gender equality. They argue in favour of a set of values (autonomy, reproductive freedom, gender equality) and argue that the respect given to a particular culture is inadequate and that respect for cultural norms is unjustified.

However, one could not help but wonder what different analytical lenses applied to this case could reveal about the lived experiences of Ikwerre women. One example springs to mind—Miranda Fricker’s (2007) concept of hermeneutical injustice, defined as “the injustice of having some significant area of one’s social experience obscured from collective understanding” (155). This is exactly the case of the Ikwerre women, who cannot conceptualize rape in marriage as it is not recognized in their culture. The adoption of Fricker’s lens could, I suspect, point not necessarily to a need to “increase an awareness of reproductive rights” but instead to the extent to which these women are systematically discredited as “epistemic subjects” and therefore prevented from being able to produce knowledge due to the absence itself of the concept of rape in marriage as a “currency exchange” in that particular economy of knowledge. This lens would reveal power hierarchies in knowledge that translate into oppressive practices in society and articulate one of the axes of biopower in reproduction.

Moving on, the paper by Petropanagos (2017), “Testicular Tissue Cryopreservation and Ethical Considerations: A Scoping Review” is a good example of “anticipatory bioethics” (Shick 2016) that discusses the ethics of cryopreservation of testicular tissue (TCCP) as a means to preserve fertility (“anticipatory bioethics” as TCCP is an increasingly common practice for cancer patients to preserve their fertility although so far scientists have not been successful in producing mature sperm from testicular tissue). Petropanagos’s scoping review reveals that the ethical issues have been analysed mainly through Beauchamp and Childress’ principlist lens in relation to four categories of individuals affected by the practice of TCCP: 1) current pediatric patients, 2) future adults, 3) future offspring, and 4) patients’ families. Petropanagos’s review identifies two key gaps in the range of ethical considerations thus identified—a lack of integration of TCCP with other aspects of healthcare (as this technology falls at the intersection of several ethical domains such as cancer care, pediatrics, reproductive ethics, and clinical research with resulting unique issues that arise out of the intersection of age, sex, gender, and disease context) and a gap in the ethical literature examining social context and meaning surrounding the value of genetic reproduction (pointing to how a discussion of kinship in both contexts would be helpful here).

Interestingly, Petropanagos notes that the range of limited ethical analysis of TCCP is the result of the emphasis on principlism as a framework for ethical analysis within the clinical setting. This approach lacks a discussion of the ethical considerations related to the social context and relationships between the four categories of individuals mentioned above and also lacks discussion of biopower as locus of control of the values shaping the development of medical technologies.

Another interesting point raised by Petropanagos’ article is that TCCP could be used for other types of patients, and these other uses need to be included in the ethical discussion of TCCP. Indeed, paraphrasing historian of medicine Ilana Lowy (2015), while technologies are initially shaped by the values and preferences of people who develop them, they can—and often are—later be modified by their users (202). For example, TCCP could be used for elite athletes or high-performance athletes who train to such an extent that it exposes their bodies to stresses that become unhealthy. Or, people could start to use TCCP as an “insurance policy” against what may happen later in life, in a similar way to what is happening to “egg freezing,” which is now being offered by companies as part of “employee benefits” insurance plans to “bank time” (Waldby 2015).

## Reflections

The papers in the *Bioethics and Biopolitics: Presents and Futures of Reproduction* symposium, taken together, articulate in different ways three points.

First, bioethics operates on two temporal dimensions: while the subject of speculative bioethics is the future, the actual point of influence is the present. It is in this sense that, as put by Schick (2016, 225) “the imagined future becomes an aspect of our present” and creates a “causal inversion”: it is the future that makes the present and not vice-versa. However, we must note how a form of “anticipatory bioethics” is not by any means “neutral” towards technologies, because addressing questions raised by future possible technologies as if they were already here (which, often, is not the case!) bypasses fundamental questions such as “what needs and problems [do] we have and which solutions, technological or otherwise, would best address them?” (229) and asks only “what should we do with the technologies that we have?” Bypassing these kinds of questions is in itself problematic, “for the ultimate purpose, desirability or even feasibility of the anticipated technologies cease to be viewed as live issues open to ethical scrutiny and public deliberation” (229). This is a point raised implicitly by Kendal’s paper on ectogenesis but also—although to a lesser extent—by Petropanagos’s paper on TCCP.

A second point raised by the papers in this symposium is that an analysis of current practices can shed light on other practices. For example, we can note how the discussion of ectogenesis in the paper by Kendal (2017) illuminates other current practices i.e. social policing of pregnant women. Currently, pregnant women are the locus of control of society as molding them shapes future generations, and they are controlled both informally through social pressure, or formally through medical guidelines or laws that regulate their behaviours during pregnancy (Meredith 2016). However, if we have ectogenesis, this locus of control moves and the questions become: what, and to what extent, should we control; and, who should decide what can be controlled? Extra-uterine gestation would offer a new locus of control for biopower.

Along similar veins, the discussion of sex selection though PGD in the paper by Browne (2017) points to an undercurrent of gender essentialism and to some of the problematic societal assumptions regarding gender. While we think we are promoting autonomy and gender equality by letting parents choose the sex of their children through PGD for “family balancing reasons,” we are instead reinforcing ideas and practices of gender essentialism which are fundamentally contrary to that gender equality. Browne argues that the practice of PGD for sex selection is not only based on a societal presumption of gender binary and gender essentialism but that it also reinforces this assumption by undermining the autonomy of parents who think they cannot enjoy the same kind of activity with a boy or with a girl.

Finally, two articles in this symposium point to the importance of adopting different theoretical frameworks in bioethics. The article by Kendal (2017) challenges explicitly the Robertsonian reproductive framework pervasive in so much work in reproductive bioethics, building on the critique of reproductive freedom operated by Catherine Mills. The article by Petropanagos (2017) on cryopreservation also points out the limitations of doing bioethics through only “one set of lenses” (Sherwin 1999), i.e. principlism. Frameworks, or lenses, in bioethics, can bring into focus different morally salient features of the same cases (Sherwin 1999, and also Pellegrino 1995). The adoption of different analytical lenses can illuminate morally salient features of a case, or of a practice, which would go unnoticed otherwise. It can also help unravel some of the assumptions and value judgments that we make in the context of some of the current practices of externalization and responsibilization of reproduction.

## Conclusions

As we know from Heisenberg’s uncertainty principle, observing a particle changes the position of the particle itself. The same, we could say, happens in bioethics, where an analysis of the futures of reproduction changes other, existing practices. Observing is intervening (Hacking 1983). That is why it is important to reflect on reproductive practices in contexts, both diachronically (looking at practices in their historical context) and synchronically (looking at practices in their different declinations in different cultures). Although “considerable analysis—both conceptual and empirical—is still required to illuminate the politics and ethics of choice in reproductive biopower today” (Mills 2015, 120), this *Bioethics and Biopolitics: Presents and Futures of Reproduction* symposium is a step in the right direction.
